# Crystal structure and Hirshfeld surface analysis of a bromo­chalcone: (*E*)-1-(3-bromo­phen­yl)-3-(2,6-di­chloro­phen­yl)prop-2-en-1-one

**DOI:** 10.1107/S205698901900104X

**Published:** 2019-01-25

**Authors:** S. Bindya, C. S. Chidan Kumar, S. Naveen, B. P. Siddaraju, Ching Kheng Quah, Md. Abu Raihan

**Affiliations:** a Department of Chemistry, Sri Jayachamarajendra College of Engineering, JSS Science and Technology University, Mysuru 570 006, Karnataka, India; b Department of Engineering Chemistry, Vidya Vikas Institute of Engineering & Technology, Visvesvaraya Technological University, Alanahally, Mysuru 570 028, India; c Department of Physics, School of Engineering and Technology, Jain University, Bangalore 562 112, India; d Department of Chemistry, Cauvery Institute of Technology, Mandya 571 402, Karnataka, India; e X-ray Crystallography Unit, School of Physics, Universiti Sains Malaysia, 11800 USM, Penang, Malaysia; f Head of TVE Department, Islamic University of Technology (IUT), Gazipur 1704, Bangladesh

**Keywords:** crystal structure, chalcone, enone bridge, *E* configuration, Cl⋯O contact, offset π–π inter­actions, Hirshfeld surface analysis, fingerprint plots

## Abstract

In the title chalcone derivative, C_15_H_9_BrCl_2_O, the aryl rings are inclined to each by 14.49 (17)°, and the configuration about the C=C bond is *E*. There is a short intra­molecular C—H⋯Cl contact present resulting in the formation of an *S*(6) ring motif. In the crystal, the shortest inter­molecular contacts are Cl⋯O contacts [3.173 (3) Å] that link the mol­ecules to form a 2_1_ helix propagating along the *b*-axis direction. The helices stack up the short crystallographic *a* axis, and are linked by offset π–π inter­actions [inter­centroid distance = 3.983 (1) Å], forming layers lying parallel to the *ab* plane. A qu­anti­fication of the inter­molecular contacts in the crystal were estimated using Hirshfeld surface analysis and two-dimensional fingerprint plots.

## Abstract 2

In the title chalcone derivative, C_15_H_9_Cl_2_BrO, the two aryl rings are inclined to each other by 14.49 (17)°, and the olefinic double bond adopts an *E* configuration. In the crystal, the only short inter­molecular contacts are Cl⋯O contacts [3.173 (3) Å] that link the mol­ecules to form a 2_1_ helix propagating along the *b*-axis direction.

## Chemical context   

Chalcones, considered to be the precursors of flavonoids and isoflavonoids, are abundant in edible plants. Chemically they consist of open-chain flavonoids in which the two aromatic rings are joined by a three-carbon, α-unsaturated carbonyl system and are described by the generic term ‘chalcone’. Chalcones are coloured compounds because of the presence of the –CO—CH=CH– chromophore, which depends on the presence of other auxochromes. Chalcones are finding applications as organic non-linear optical materials (NLO) because of their good SHG conversion efficiencies (Chandra Shekhara Shetty *et al.*, 2016 ▸; Raghavendra *et al.*, 2017 ▸). In view of this inter­est we have synthesized the title chalcone derivative and report herein on its crystal structure and Hirshfeld surface analysis.
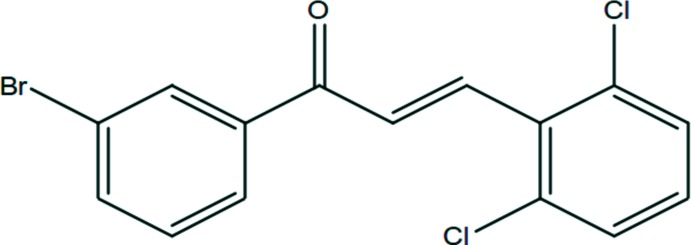


## Structural commentary   

**Figure 1:**
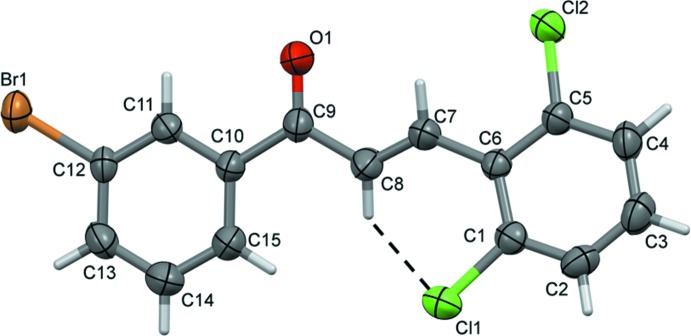
The mol­ecular structure of the title compound, with atom labelling. Displacement ellipsoids are drawn at the 50% probability level. The intra­molecular C—H⋯Cl hydrogen bond (Table 1 ▸) is shown as a dashed line.

The mol­ecular structure of the title compound is shown in Fig. 1 ▸. It comprises two aromatic rings (2,6-di­chloro­phenyl and 3-bromo­phen­yl) linked by the C7=C8—C9(=O1)—C10 enone bridge. The bond lengths and bond angles are normal and the mol­ecular conformation is characterized by a dihedral angle of 14.49 (17)° between the mean planes of the two aromatic rings. The olefinic double bond [C7=C8 = 1.286 (5) Å] is in an *E* configuration. There is a short intra­molecular C—H⋯Cl contact present resulting in the formation of an *S*(6) ring motif (Fig. 1 ▸ and Table 1 ▸). The unsaturated keto group is in a *syn*-periplanar conformation with respect to the olefinic double bond, which is evident from the O1—C9—C8—C7 torsion angle of 10.9 (6)°. The *trans* conformation of the C=C double bond in the central enone group is confirmed by the C6—C7—C8=C9 torsion angle of −179.8 (3)°. The bond angles O1—C9—C10 [120.4 (3)°], O1—C9—C8 [119.9 (3)°] and C9—C8—C7 [123.9 (4)°] about C9 indicate that this carbon atom is in a distorted trigonal–planar conformation.

## Supra­molecular features   

**Figure 2:**
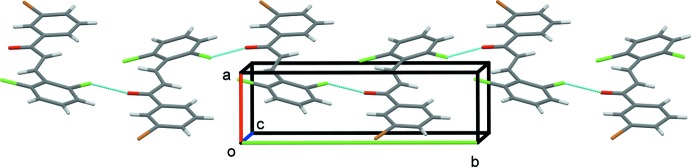
A partial view along the *c* axis of the crystal packing of the title compound. The inter­molecular Cl⋯O inter­actions are shown as dashed lines.

**Figure 3:**
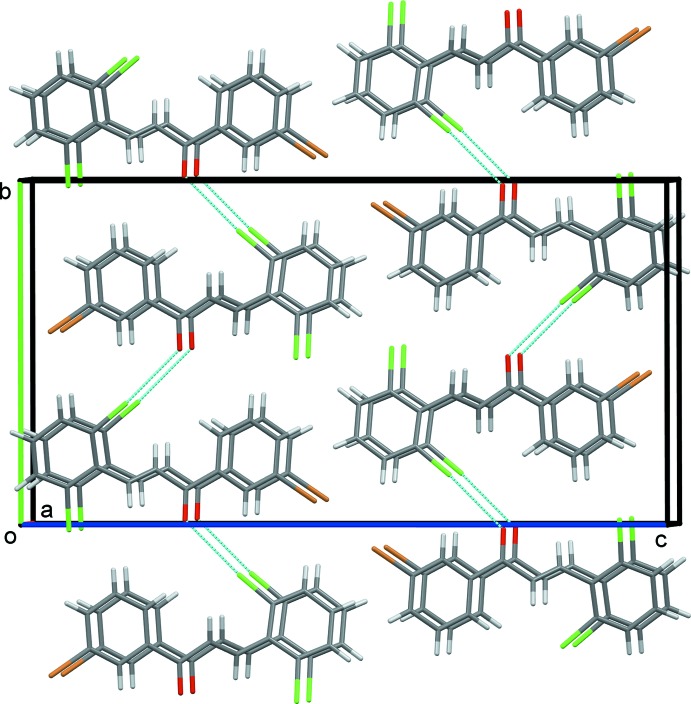
A view along the *a* axis of the crystal packing of the title compound. The inter­molecular Cl⋯O inter­actions are shown as dashed lines.

In the crystal, the mol­ecules stack along the short crystallographic *a* axis. The shortest inter­molecular contacts are Cl⋯O1^i^ contacts [3.173 (3) Å; symmetry code (i): −*x* + 2, *y* + 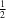, −*z* + 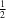] that link the mol­ecules to form 2_1_ helices propagating along the *b*-axis direction (Fig. 2 ▸). The helices are linked by offset π–π inter­actions, forming undulating layers lying parallel to the *ab* plane, see Fig. 3 ▸ [*Cg*1⋯*Cg*1^ii^ = 3.983 (2) Å, α = 0.0 (2)°, β = 24.7°, inter­planar distance = 3.6193 (14) Å, offset 1.66 Å; *Cg*2⋯*Cg*2^iii^ = 3.984 (2) Å, α = 0.0 (2) °, β = 24.8 °, offset = 1.67 Å; *Cg*1 and *Cg*2 are the centroids of C1–C6 and C10–C15 rings, respectively; symmetry codes: (ii) *x* − 1, *y*, *z*; (iii) *x* + 1, *y*, *z*].

## Hirshfeld surface analysis   

**Figure 4:**
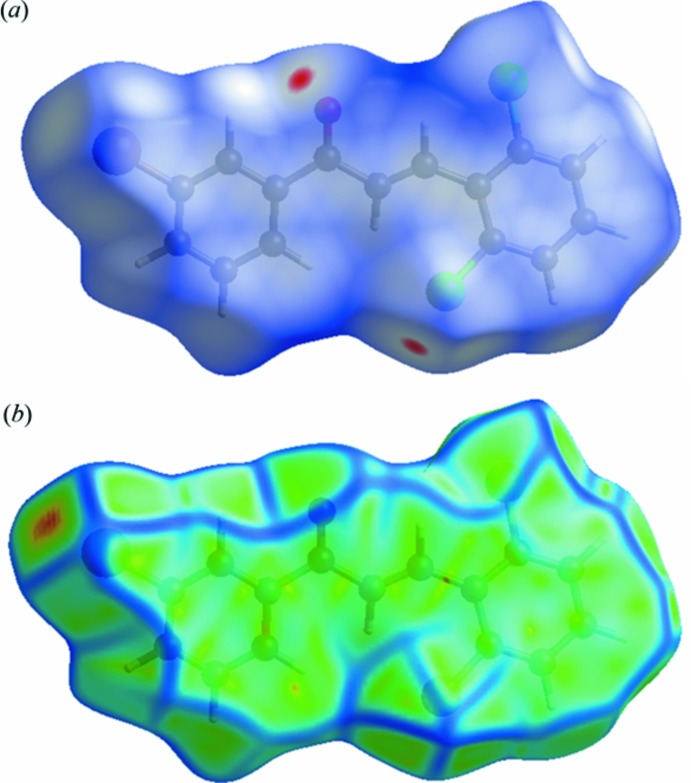
A view of the three-dimensional Hirshfeld surface of the title compound mapped over (*a*) *d*_norm_ and (*b*) curvedness.

Hirshfeld surfaces and fingerprint plots were generated for the title compound using *CrystalExplorer* (Wolff *et al.*, 2012 ▸). Hirshfeld surfaces enable the visualization of inter­molecular inter­actions by different colours and colour intensity, representing short or long contacts and indicating the relative strength of the inter­actions. Fig. 4 ▸ *a* shows the Hirshfeld surfaces mapped over *d*_norm_, while Fig. 4 ▸ *b* shows the Hirshfeld surfaces mapped over curvedness. In Fig. 4 ▸ *a*, the red spots near atoms Cl1 and O1 result from the Cl⋯O inter­actions, which play a significant role in the mol­ecular packing of the title compound (Figs. 2 ▸ and 3 ▸), and the Cl⋯H/H⋯Cl and O⋯H/H⋯O contacts. The curvedness plot (Fig. 4 ▸ *b*) shows an extensive flat surface characteristic of planar stacking – see the *Supra­molecular features* section above.

**Figure 5:**
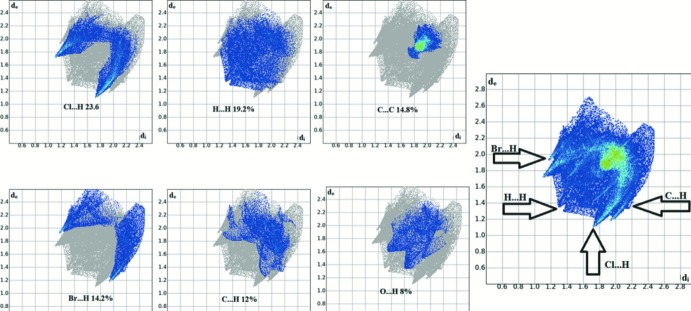
Two-dimensional fingerprint plots of the title compound showing the percentage contributions of all inter­actions, and the most significant individual types of inter­actions.

The overall two-dimensional fingerprint plot (McKinnon *et al.*, 2007 ▸), for the title compound and those delineated into Cl⋯H/H⋯Cl, H⋯H, C⋯C, Br⋯H/H⋯Br, C⋯H/H⋯C, O⋯H/H⋯O contacts are illustrated in Fig. 5 ▸; the most significant contributions from the different inter­atomic contacts to the Hirshfeld surfaces are as follows: Cl⋯H (23.6%), H⋯H (19.2%), C⋯C (14.8%), Br⋯H (14.2%), C⋯H (12%) and O⋯H (8%). Other inter­molecular contacts contribute less than 5% to the Hirshfeld surface mapping. Inter­estingly, the Cl⋯O inter­actions (Fig. 2 ▸) make a contribution of only 2.2% to the Hirshfeld surface.

## Database survey   

A search of the Cambridge Structural Database (CSD, Version 5.40, last update November 2018; Groom *et al.*, 2016 ▸) using 1-(3-bromo­phen­yl)-3-phenyl­prop-2-en-1-one as the main skeleton revealed the presence of 12 structures (see supporting information), including 1-(3-bromo­phen­yl)-3-phenyl­prop-2-en-1-one itself (CSD refcode CICLUW; Rosli *et al.*, 2007 ▸). The other structures closest to the title compound with a second halogen-substituted phenyl ring are: 1-(3-bromo­phen­yl)-3-(4-chloro­phen­yl)prop-2-en-1-one (VIDFEU; Teh *et al.*, 2007 ▸), 1-(3-bromo­phen­yl)-3-(3-fluoro­phen­yl)prop-2-en-1-one (GASBEK; Rajendraprasad *et al.*, 2017 ▸), and 1-(3-bromo­phen­yl)-3-(4-fluoro­phen­yl)prop-2-en-1-one (OBIYUW; Ekbote *et al.*, 2017 ▸). In these four compounds, the two benzene rings are inclined to each other by *ca* 49.93, 46.71, 48.92 and 47.74°, respectively. The same dihedral angle in the title compound is only 14.49 (17)° because of the presence of the intra­molecular C—H⋯Cl hydrogen bond, as shown in Fig. 1 ▸ (Table 1 ▸).

## Synthesis and crystallization   

The title compound was synthesized according to a reported procedure (Chidan Kumar *et al.*, 2014 ▸). 1-(3-Bromo­phen­yl)ethanone (0.01 mol) and 2,6-di­chloro­benzaldehyde (0.01 mol) were dissolved in 20 ml of methanol. A catalytic amount of NaOH was added dropwise with vigorous stirring. The reaction mixture was stirred for about 3 h at room temperature. The crude product was filtered, washed several times with distilled water and recrystallized from methanol. On slow evaporation of the solvent, colourless plate-like crystals of the title compound were obtained (m.p. 327–330 K).

## Refinement   

Crystal data, data collection and structure refinement details are summarized in Table 2 ▸. The C-bound H atoms were positioned geometrically (C—H = 0.95 Å) and refined using a riding model with *U*_iso_(H) = 1.2*U*_eq_(C).

## Supplementary Material

Crystal structure: contains datablock(s) global, I. DOI: 10.1107/S205698901900104X/qm2132sup1.cif

Structure factors: contains datablock(s) I. DOI: 10.1107/S205698901900104X/qm2132Isup2.hkl

Supporting information file. DOI: 10.1107/S205698901900104X/qm2132Isup3.cml

Details of CSD search. DOI: 10.1107/S205698901900104X/qm2132sup4.pdf

CCDC reference: 1036741

Additional supporting information:  crystallographic information; 3D view; checkCIF report

## Figures and Tables

**Table 1:** Hydrogen-bond geometry (Å, °)

*D*—H⋯*A*	*D*—H	H⋯*A*	*D*⋯*A*	*D*—H⋯*A*
C8—H8*A*⋯Cl1	0.93	2.54	3.128 (4)	122

**Table 2:** Experimental details

Crystal data	
Chemical formula	C_15_H_9_BrCl_2_O
*M*_r_	356.02
Crystal system, space group	Monoclinic, *P*2_1_/*c*
Temperature (K)	294
*a*, *b*, *c* (Å)	3.9834 (7), 13.471 (2), 25.661 (4)
β (°)	92.736 (4)
*V* (Å^3^)	1375.4 (4)
*Z*	4
Radiation type	Mo *K*α
μ (mm^−1^)	3.36
Crystal size (mm)	0.47 × 0.14 × 0.05

Data collection	
Diffractometer	Bruker APEXII DUO CCD area-detector
Absorption correction	Multi-scan (*SADABS*; Bruker, 2012 ▸)
*T*_min_, *T*_max_	0.303, 0.842
No. of measured, independent and observed [*I* > 2σ(*I*)] reflections	10857, 3242, 2260
*R*_int_	0.037
(sin θ/λ)_max_ (Å^−1^)	0.657

Refinement	
*R*[*F* ^2^ > 2σ(*F* ^2^)], *wR*(*F* ^2^), *S*	0.041, 0.124, 1.04
No. of reflections	3242
No. of parameters	172
H-atom treatment	H atoms treated by a mixture of independent and constrained refinement
Δρ_max_, Δρ_min_ (e Å^−3^)	0.47, −0.33

Computer programs: *APEX2* and *SAINT* (Bruker, 2012 ▸), *SHELXS97* and *SHELXL97* (Sheldrick, 2008 ▸), *Mercury* (Macrae *et al.*, 2008 ▸), *PLATON* (Spek, 2009 ▸) and *publCIF* (Westrip, 2010 ▸).

## supplementary crystallographic information

### Crystal data

**Table:**

C_15_H_9_BrCl_2_O	*F*(000) = 704
*M**_r_* = 356.02	*D*_x_ = 1.719 Mg m^−^^3^
Monoclinic, *P*2_1_/*c*	Mo *K*α radiation, λ = 0.71073 Å
Hall symbol: -P 2ybc	Cell parameters from 2260 reflections
*a* = 3.9834 (7) Å	θ = 1.6–27.8°
*b* = 13.471 (2) Å	µ = 3.36 mm^−^^1^
*c* = 25.661 (4) Å	*T* = 294 K
β = 92.736 (4)°	Plate, colourless
*V* = 1375.4 (4) Å^3^	0.47 × 0.14 × 0.05 mm
*Z* = 4	

### Data collection

**Table:**

Bruker APEXII DUO CCD area-detector diffractometer	3242 independent reflections
Radiation source: Rotating Anode	2260 reflections with *I* > 2σ(*I*)
Graphite monochromator	*R*_int_ = 0.037
Detector resolution: 18.4 pixels mm^-1^	θ_max_ = 27.8°, θ_min_ = 1.6°
φ and ω scans	*h* = −5→5
Absorption correction: multi-scan (SADABS; Bruker, 2012)	*k* = −17→15
*T*_min_ = 0.303, *T*_max_ = 0.842	*l* = −31→33
10857 measured reflections	

### Refinement

**Table:**

Refinement on *F*^2^	Primary atom site location: structure-invariant direct methods
Least-squares matrix: full	Secondary atom site location: difference Fourier map
*R*[*F*^2^ > 2σ(*F*^2^)] = 0.041	Hydrogen site location: inferred from neighbouring sites
*wR*(*F*^2^) = 0.124	H atoms treated by a mixture of independent and constrained refinement
*S* = 1.04	*w* = 1/[σ^2^(*F*_o_^2^) + (0.0691*P*)^2^] where *P* = (*F*_o_^2^ + 2*F*_c_^2^)/3
3242 reflections	(Δ/σ)_max_ = 0.001
172 parameters	Δρ_max_ = 0.47 e Å^−^^3^
0 restraints	Δρ_min_ = −0.33 e Å^−^^3^

### Special details

**Table:**

Geometry. Bond distances, angles etc. have been calculated using the rounded fractional coordinates. All su's are estimated from the variances of the (full) variance-covariance matrix. The cell esds are taken into account in the estimation of distances, angles and torsion angles
Refinement. Refinement on F^2^ for ALL reflections except those flagged by the user for potential systematic errors. Weighted R-factors wR and all goodnesses of fit S are based on F^2^, conventional R-factors R are based on F, with F set to zero for negative F^2^. The observed criterion of F^2^ > 2sigma(F^2^) is used only for calculating -R-factor-obs etc. and is not relevant to the choice of reflections for refinement. R-factors based on F^2^ are statistically about twice as large as those based on F, and R-factors based on ALL data will be even larger.

### Fractional atomic coordinates and isotropic or equivalent isotropic displacement parameters (Å^2^)

**Table:**

	*x*	*y*	*z*	*U*_iso_*/*U*_eq_	
Br1	0.05103 (10)	0.56559 (3)	0.06384 (1)	0.0533 (2)	
Cl1	1.1724 (3)	0.85319 (6)	0.33444 (4)	0.0585 (3)	
Cl2	1.1077 (3)	0.48639 (7)	0.42380 (4)	0.0629 (4)	
O1	0.6757 (10)	0.5127 (2)	0.25232 (11)	0.0795 (13)	
C1	1.2349 (8)	0.7699 (2)	0.38553 (13)	0.0400 (10)	
C2	1.3925 (9)	0.8068 (3)	0.43034 (14)	0.0482 (11)	
C3	1.4602 (10)	0.7456 (3)	0.47251 (15)	0.0551 (12)	
C4	1.3682 (9)	0.6477 (3)	0.46994 (13)	0.0489 (11)	
C5	1.2137 (9)	0.6115 (2)	0.42448 (13)	0.0411 (10)	
C6	1.1375 (8)	0.6695 (2)	0.38034 (12)	0.0344 (9)	
C7	0.9826 (9)	0.6237 (2)	0.33364 (13)	0.0423 (11)	
C8	0.8005 (9)	0.6605 (3)	0.29567 (13)	0.0464 (11)	
C9	0.6625 (9)	0.6020 (3)	0.25107 (13)	0.0433 (11)	
C10	0.5026 (8)	0.6542 (2)	0.20468 (12)	0.0386 (10)	
C11	0.3737 (8)	0.5979 (2)	0.16350 (12)	0.0386 (10)	
C12	0.2288 (8)	0.6434 (2)	0.12041 (12)	0.0377 (10)	
C13	0.2083 (10)	0.7454 (3)	0.11687 (14)	0.0512 (12)	
C14	0.3403 (11)	0.8019 (3)	0.15749 (15)	0.0572 (14)	
C15	0.4858 (10)	0.7575 (3)	0.20135 (15)	0.0494 (11)	
H2A	1.45350	0.87340	0.43210	0.0580*	
H3A	1.56810	0.77070	0.50260	0.0660*	
H4A	1.40930	0.60610	0.49850	0.0590*	
H7A	1.02250	0.55590	0.33080	0.0510*	
H8A	0.75310	0.72810	0.29640	0.0560*	
H11A	0.38530	0.52900	0.16500	0.0460*	
H13A	0.10710	0.77540	0.08750	0.0620*	
H14A	0.33120	0.87080	0.15540	0.0680*	
H15A	0.57290	0.79640	0.22870	0.0590*	

### Atomic displacement parameters (Å^2^)

**Table:**

	*U*^11^	*U*^22^	*U*^33^	*U*^12^	*U*^13^	*U*^23^
Br1	0.0585 (3)	0.0625 (3)	0.0375 (2)	−0.0001 (2)	−0.0130 (2)	−0.0037 (2)
Cl1	0.0795 (7)	0.0395 (5)	0.0556 (6)	−0.0046 (4)	−0.0054 (5)	0.0095 (4)
Cl2	0.0904 (8)	0.0433 (5)	0.0526 (6)	−0.0130 (5)	−0.0208 (5)	0.0112 (4)
O1	0.140 (3)	0.0403 (16)	0.0538 (17)	0.0047 (16)	−0.0406 (19)	−0.0011 (13)
C1	0.0414 (18)	0.0401 (18)	0.0388 (18)	0.0025 (14)	0.0054 (15)	−0.0002 (14)
C2	0.048 (2)	0.0391 (19)	0.057 (2)	−0.0045 (15)	−0.0027 (18)	−0.0106 (17)
C3	0.055 (2)	0.063 (2)	0.046 (2)	−0.0017 (19)	−0.0109 (18)	−0.0121 (19)
C4	0.054 (2)	0.059 (2)	0.0326 (18)	0.0010 (17)	−0.0085 (16)	0.0005 (16)
C5	0.0442 (18)	0.0386 (18)	0.0397 (18)	−0.0044 (14)	−0.0064 (15)	0.0003 (15)
C6	0.0350 (16)	0.0346 (16)	0.0334 (16)	0.0022 (12)	−0.0016 (13)	−0.0028 (13)
C7	0.052 (2)	0.0357 (17)	0.0386 (18)	−0.0035 (14)	−0.0037 (16)	0.0023 (14)
C8	0.056 (2)	0.0400 (19)	0.0422 (19)	0.0092 (16)	−0.0095 (17)	−0.0037 (15)
C9	0.051 (2)	0.045 (2)	0.0334 (18)	0.0050 (15)	−0.0044 (15)	−0.0037 (15)
C10	0.0458 (19)	0.0355 (17)	0.0337 (17)	0.0019 (14)	−0.0057 (14)	−0.0033 (13)
C11	0.0445 (18)	0.0369 (17)	0.0346 (17)	0.0044 (14)	0.0028 (14)	0.0011 (14)
C12	0.0393 (17)	0.047 (2)	0.0262 (15)	0.0021 (14)	−0.0030 (13)	−0.0013 (14)
C13	0.062 (2)	0.052 (2)	0.039 (2)	0.0088 (17)	−0.0042 (17)	0.0087 (17)
C14	0.078 (3)	0.041 (2)	0.052 (2)	0.0057 (18)	−0.002 (2)	0.0036 (17)
C15	0.065 (2)	0.0416 (19)	0.0409 (19)	0.0018 (17)	−0.0048 (17)	−0.0043 (16)

### Geometric parameters (Å, º)

**Table:**

Br1—C12	1.900 (3)	C10—C15	1.396 (5)
Cl1—C1	1.735 (3)	C11—C12	1.368 (4)
Cl2—C5	1.737 (3)	C12—C13	1.379 (5)
O1—C9	1.205 (5)	C13—C14	1.375 (5)
C1—C2	1.376 (5)	C14—C15	1.378 (6)
C1—C6	1.412 (4)	C2—H2A	0.9300
C2—C3	1.377 (5)	C3—H3A	0.9300
C3—C4	1.370 (6)	C4—H4A	0.9300
C4—C5	1.382 (5)	C7—H7A	0.9300
C5—C6	1.397 (4)	C8—H8A	0.9300
C6—C7	1.458 (4)	C11—H11A	0.9300
C7—C8	1.286 (5)	C13—H13A	0.9300
C8—C9	1.474 (5)	C14—H14A	0.9300
C9—C10	1.498 (5)	C15—H15A	0.9300
C10—C11	1.380 (4)		
			
Cl1—C1—C2	116.2 (2)	C11—C12—C13	121.4 (3)
Cl1—C1—C6	121.3 (2)	C12—C13—C14	118.8 (3)
C2—C1—C6	122.5 (3)	C13—C14—C15	120.7 (4)
C1—C2—C3	120.3 (4)	C10—C15—C14	120.0 (4)
C2—C3—C4	119.8 (4)	C1—C2—H2A	120.00
C3—C4—C5	119.2 (3)	C3—C2—H2A	120.00
Cl2—C5—C4	116.7 (3)	C2—C3—H3A	120.00
Cl2—C5—C6	119.4 (2)	C4—C3—H3A	120.00
C4—C5—C6	123.9 (3)	C3—C4—H4A	120.00
C1—C6—C5	114.3 (3)	C5—C4—H4A	120.00
C1—C6—C7	125.9 (3)	C6—C7—H7A	114.00
C5—C6—C7	119.8 (3)	C8—C7—H7A	114.00
C6—C7—C8	131.4 (3)	C7—C8—H8A	118.00
C7—C8—C9	123.9 (4)	C9—C8—H8A	118.00
O1—C9—C8	119.9 (3)	C10—C11—H11A	120.00
O1—C9—C10	120.4 (3)	C12—C11—H11A	120.00
C8—C9—C10	119.6 (3)	C12—C13—H13A	121.00
C9—C10—C11	118.6 (3)	C14—C13—H13A	121.00
C9—C10—C15	122.3 (3)	C13—C14—H14A	120.00
C11—C10—C15	119.1 (3)	C15—C14—H14A	120.00
C10—C11—C12	120.0 (3)	C10—C15—H15A	120.00
Br1—C12—C11	119.9 (2)	C14—C15—H15A	120.00
Br1—C12—C13	118.7 (2)		
			
Cl1—C1—C2—C3	−178.4 (3)	C7—C8—C9—O1	10.9 (6)
C6—C1—C2—C3	−0.3 (5)	C7—C8—C9—C10	−169.8 (3)
Cl1—C1—C6—C5	178.3 (2)	O1—C9—C10—C11	−0.5 (5)
Cl1—C1—C6—C7	0.8 (5)	O1—C9—C10—C15	−179.2 (4)
C2—C1—C6—C5	0.2 (5)	C8—C9—C10—C11	−179.7 (3)
C2—C1—C6—C7	−177.3 (3)	C8—C9—C10—C15	1.6 (5)
C1—C2—C3—C4	−0.4 (6)	C9—C10—C11—C12	−179.4 (3)
C2—C3—C4—C5	1.2 (6)	C15—C10—C11—C12	−0.7 (5)
C3—C4—C5—Cl2	179.4 (3)	C9—C10—C15—C14	179.1 (4)
C3—C4—C5—C6	−1.3 (6)	C11—C10—C15—C14	0.4 (5)
Cl2—C5—C6—C1	179.9 (3)	C10—C11—C12—Br1	−179.9 (2)
Cl2—C5—C6—C7	−2.4 (4)	C10—C11—C12—C13	0.2 (5)
C4—C5—C6—C1	0.6 (5)	Br1—C12—C13—C14	−179.2 (3)
C4—C5—C6—C7	178.3 (3)	C11—C12—C13—C14	0.7 (5)
C1—C6—C7—C8	−26.8 (6)	C12—C13—C14—C15	−1.0 (6)
C5—C6—C7—C8	155.8 (4)	C13—C14—C15—C10	0.5 (6)
C6—C7—C8—C9	−179.8 (3)		

### Hydrogen-bond geometry (Å, º)

**Table:**

*D*—H···*A*	*D*—H	H···*A*	*D*···*A*	*D*—H···*A*
C8—H8*A*···Cl1	0.93	2.54	3.128 (4)	122
