# Supplementary material for: Crystal structure and Hirshfeld surface analysis of a bromo­chalcone: (E)-1-(3-bromo­phen­yl)-3-(2,6-di­chloro­phen­yl)prop-2-en-1-one
Source: Acta Crystallogr E Crystallogr Commun. 2019 Jan 25;75(Pt 2):264–7. doi: 10.1107/S205698901900104X (PMC6362637; doi:10.1107/S205698901900104X)

Search Overview

Search:	search2
Date/Time done:	Tue Jan 22 16:03:13 2019
Database(s):	CSD version 5.40 (November 2018)
Restriction Info:	No refcode restrictions applied
Filters:	None
Percentage Completed:	100%
Number of Hits:	31

Single query used. Search found structures that:

match

Query 1

Query 1

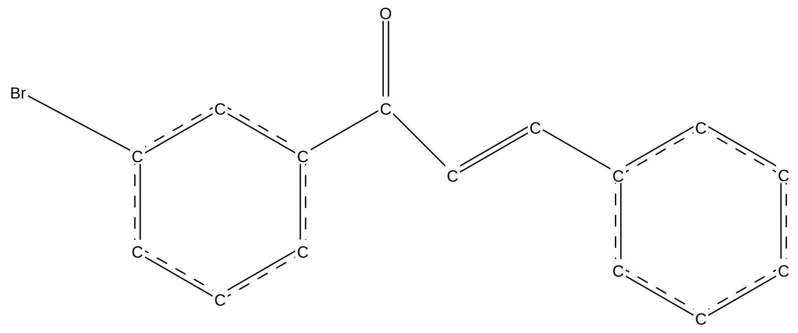

Search: search2 (Tue Jan 22 16:03:13 2019): Hit 1

AJUFAO

Reference:	E.D.Dsilva, D.Narayan Rao, Reji Philip, R.J.Butcher, Rajnikant, S.M.Dharmaprakash (2011) *Physica B: Condensed Matter(Amsterdam)* ,**406,**2206
Formula:	C_{16} H_{13} Br_1 O_1 S_1
Compound Name:	(2E)-3-[4-(Methylsulfanyl)phenyl]-1-(3-bromophenyl)prop-2-en-1-one

Space Group:	Pbca	***Cell:***	***a***	13.941(1)	***b***	5.802(0)	***c***	33.496(4)
Space Group No.:	61	***(Å,°)***	α	90.00	β	90.00	γ	90.00

R-Factor (%):	0.00	***Temperature(K):***	100	***Density(g/cm³):***	1.634

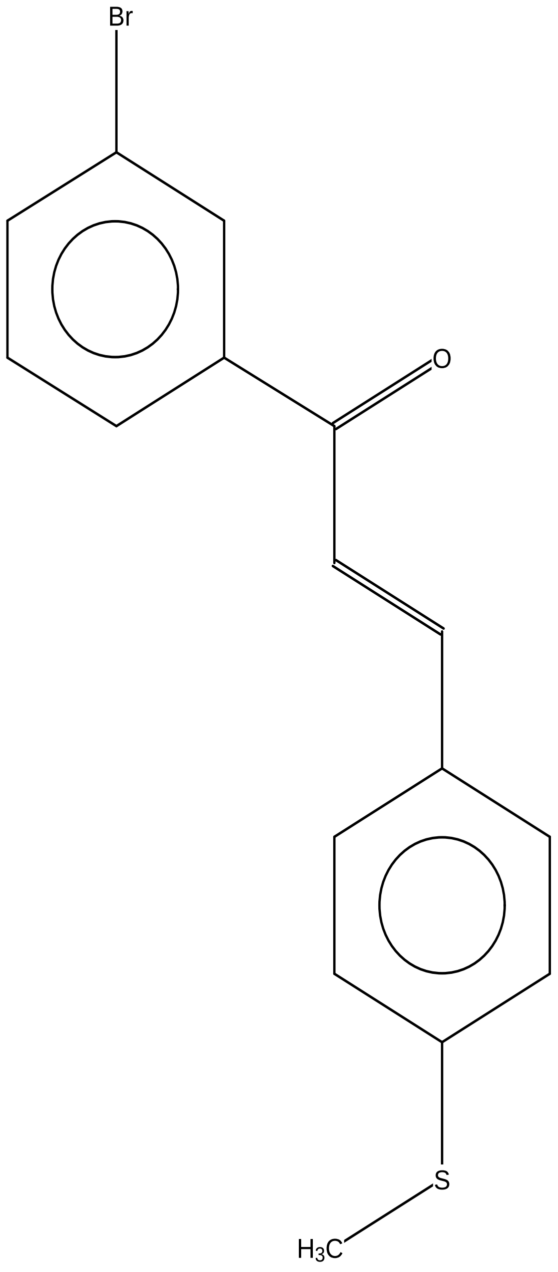

Parameters

Fragment 1

ANG1 (A) 50.030

Search: search2 (Tue Jan 22 16:03:13 2019): Hit 2

CICLUW

Reference:	M.M.Rosli, P.S.Patil, Hoong-Kun Fun, I.A.Razak, S.M.Dharmaprakash (2007) *Acta Crystallogr.,Sect.E:Struct.Rep.Online* ,**63,**o2501
Formula:	C_{15} H_{11} Br_1 O_1
Compound Name:	(2E)-1-(3-Bromophenyl)-3-phenylprop-2-en-1-one

Space Group:	P-1	***Cell:***	***a***	5.825(0)	***b***	7.633(0)	***c***	13.337(0)
Space Group No.:	2	***(Å,°)***	α	83.05(0)	β	89.92(0)	γ	87.38(0)

R-Factor (%): 2.59 ***Temperature(K):*** 100 ***Density(g/cm³):*** 1.622

Parameters

Fragment 1

ANG1 (A) 49.933

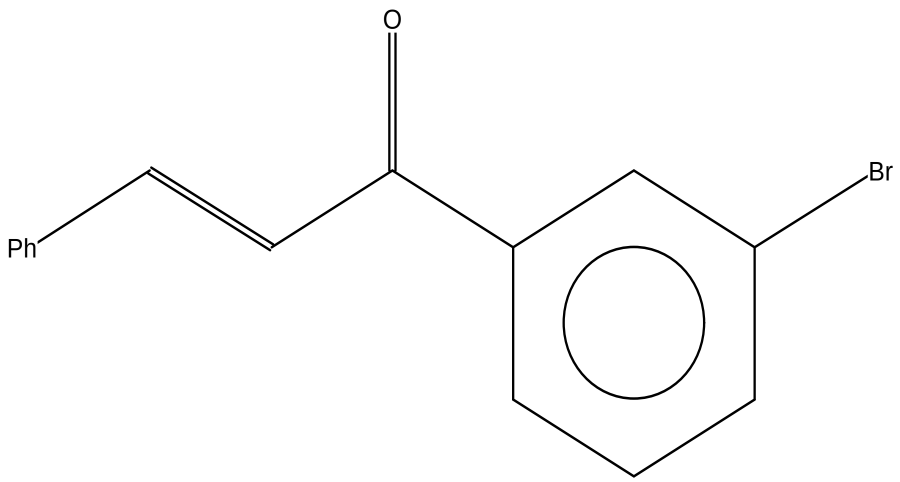

Search: search2 (Tue Jan 22 16:03:13 2019): Hit 3

GASBEK

Reference:	S.Rajendraprasad, C.S.Chidan Kumar, C.K.Quah, S.Chandraju, N.K.Lokanath, S.Naveen, I.Warad (2017) *IUCrData* ,**2,** x170379
Formula:	C_{15} H_{10} Br_1 F_1 O_1
Compound Name:	(E)-1-(3-Bromophenyl)-3-(3-fluorophenyl)prop-2-en-1-one

Space Group:	P21/n	***Cell:***	***a*** 7.603(0)		***b*** 5.928(0)		***c*** 27.600(3)
Space Group No.:	14	***(Å,°)***	α 90.00		β 93.18(0)		γ 90.00
R-Factor (%):	3.35	***Temperature(K):***	100		***Density(g/cm³):***		1.632

Parameters

Fragment 1

ANG1 (A) 48.923

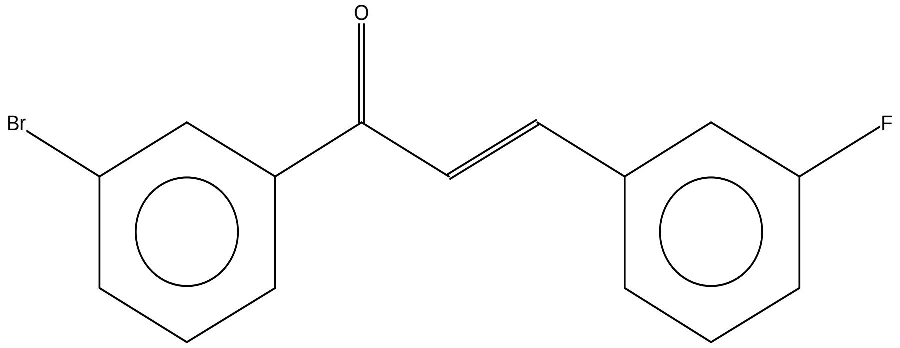

Search: search2 (Tue Jan 22 16:03:13 2019): Hit 4

JOFKAR

Reference:	H.-K.Fun, S.Chantrapromma, P.S.Patil, S.M.Dharmaprakash (2008) *Acta Crystallogr.,Sect.E:Struct.Rep.Online* ,**64,**o1356
Formula:	C_{17} H_{15} Br_1 O_2
Compound Name:	1-(3-Bromophenyl)-3-(4-ethoxyphenyl)prop-2-en-1-one

Space Group:	P21	***Cell:***	***a***	4.052(0)	***b***	9.650(0)	***c***	17.912(0)
Space Group No.:	4	***(Å,°)***	α	90.00	β	92.40(0)	γ	90.00

R-Factor (%):	3.43	***Temperature(K):***	100	***Density(g/cm^3):***	1.572

Parameters

Fragment 1
ANG1 (A) 10.087

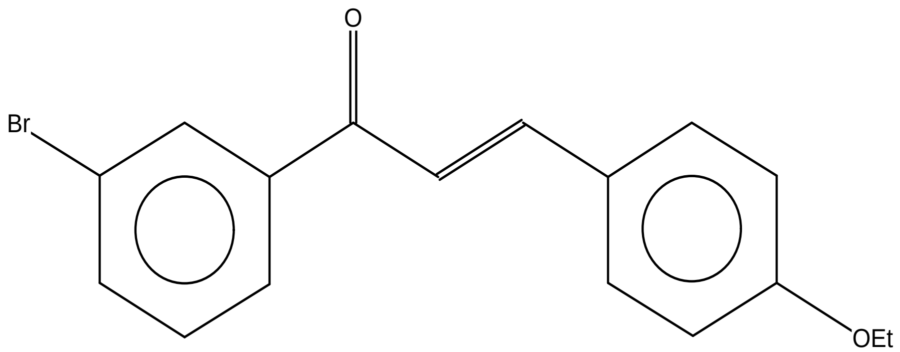

Search: search2 (Tue Jan 22 16:03:13 2019): Hit 5

LAQWUX

Reference:	C.A.Escobar, A.Trujillo, J.A.K.Howard, M.Fuentealba<br>(2012) *Acta Crystallogr.,Sect.E:Struct.Rep.Online* ,**68,**o887
Formula:	C_{17} H_{15} Br_1 O_3
Compound Name:	(E)-1-(3-Bromophenyl)-3-(3,4-dimethoxyphenyl)prop-2-en-1-one

Space Group:	P21/c	***Cell:***	***a***	12.795(0)	***b***	3.937(0)	***c***	29.821(1)
Space Group No.:	14	***(Å,°)***	α	90.00	β	109.22(0)	γ	90.00

R-Factor (%): 4.05 ***Temperature(K):*** 120 ***Density(g/cm^3):*** 1.626

Parameters

Fragment 1

ANG1 (A) 26.582

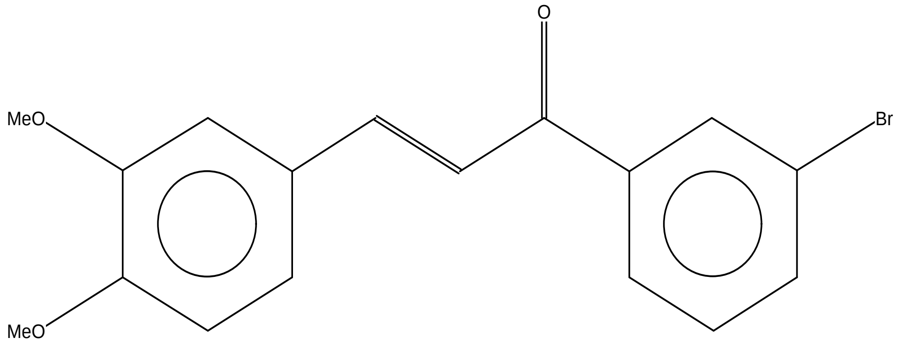

Search: search2 (Tue Jan 22 16:03:13 2019): Hit 6

OBIYUW

Reference:	Anusha Ekbote, P.S.Patil, S.R.Maidur, Tze Shyang Chia, Ching Kheng Quah (2017) *Dyes Pigm.* ,**139,**720
Formula:	C_{15} H_{10} Br_1 F_1 O_1
Compound Name:	1-(3-bromophenyl)-3-(4-fluorophenyl)prop-2-en-1-one

Space Group:	P-1	***Cell:***	***a*** 5.865(0)	***b*** 7.417(0)	***c*** 14.032(0)
Space Group No.:	2	***(Å,°)***	α 90.81(0)	β 97.03(0)	γ 92.57(0)
R-Factor (%):	3.53	***Temperature(K):***	100	***Density(g/cm³):***	1.675

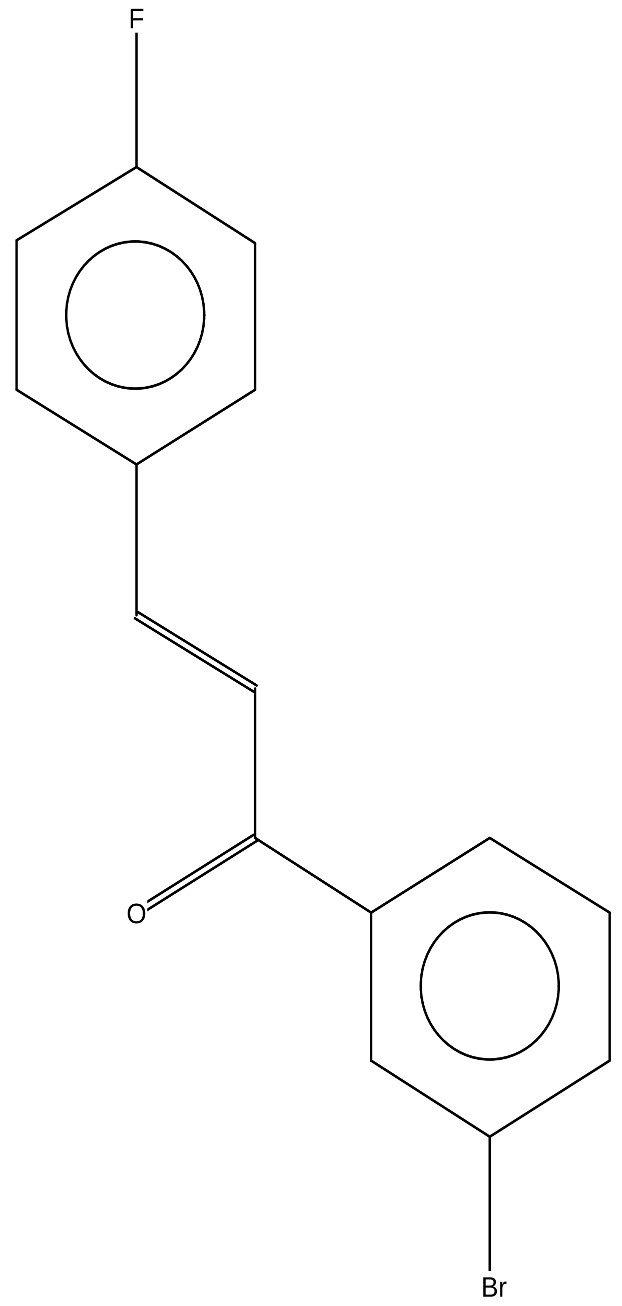

Parameters

Fragment 1
ANG1 (A) 47.741

Search: search2 (Tue Jan 22 16:03:13 2019): Hit 7

PUGVAP

Reference:	G.Dutkiewicz, C.S.C.Kumar, H.S.Yathirajan, B.Narayana, M.Kubicki (2009) *Acta Crystallogr.,Sect.E:Struct.Rep.Online* ,**65,**o2856
Formula:	C_{21} H_{15} Br_1 O_1
Compound Name:	(E)-3-(Biphenyl-4-yl)-1-(3-bromophenyl)prop-2-en-1-one

Space Group:	Pca21	***Cell:***	***a*** 6.092(1)	***b*** 7.295(1)	***c*** 36.619(2)		
Space Group No.:	29	***(Å,°)***	α 90.00	β 90.00	γ 90.00		
R-Factor (%):	3.45	***Temperature(K):***	295	***Density(g/cm^3):***	1.483		

Parameters

Fragment 1

ANG1 (A) 47.080

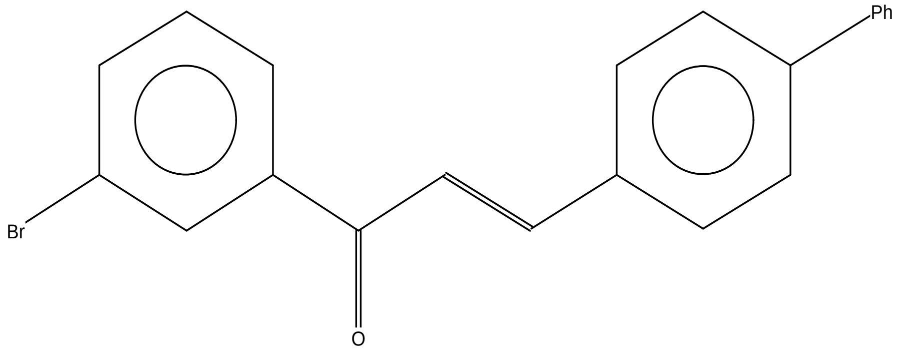

Search: search2 (Tue Jan 22 16:03:13 2019): Hit 8

VABXIH

Reference:	J.P.Jasinski, R.J.Butcher, C.S.Chidan Kumar, H.S.Yathirajan, A.N.Mayekar (2010) *Acta Crystallogr.,Sect.E:Struct.Rep.Online* ,**66,**o2936
Formula:	$C_{17}\ H_{14}\ Br_{1}\ N_{1}\ O_{5}$
Compound Name:	(2E)-1-(3-Bromophenyl)-3-(4,5-dimethoxy-2-nitrophenyl)prop-2-en-1-one

Space Group:	P212121	***Cell:***	***a*** 6.855(0)	***b*** 8.321(0)	***c*** 27.151(0)
Space Group No.:	19	***(Å,°)***	α 90.00	β 90.00	γ 90.00

R-Factor (%): 3.22 ***Temperature(K):*** 123 ***Density(g/cm³):*** 1.682

Parameters

Fragment 1

ANG1 (A) 15.197

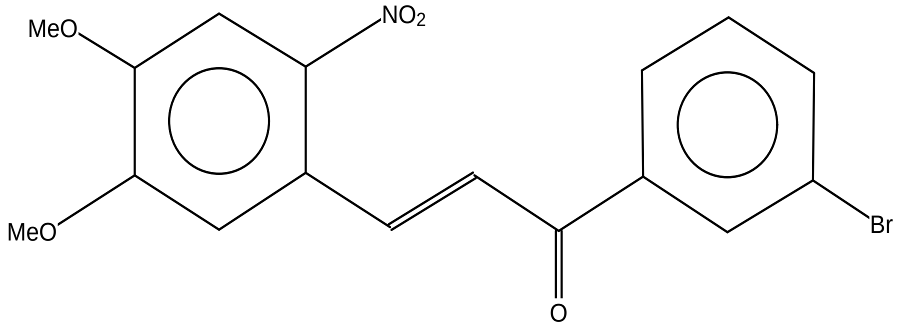

Search: search2 (Tue Jan 22 16:03:13 2019): Hit 9

VIDFEU

Reference:	J.Bee-Jan Teh, P.S.Patil, H.-K.Fun, Y.E.Satheesh, I.A.Razak, S.M.Dharmaprakash (2007) *Acta Crystallogr.,Sect.E:Struct.Rep.Online* ,**63,**o1844
Formula:	C_{15} H_{10} Br_1 Cl_1 O_1
Compound Name:	(2E)-1-(3-Bromophenyl)-3-(4-chlorophenyl)prop-2-en-1-one

Space Group:	P-1	***Cell:***	***a***	5.916(0)	***b***	7.264(0)	***c***	14.663(0)
Space Group No.:	2	***(Å,°)***	α	86.45(0)	β	83.24(0)	γ	87.86(0)

R-Factor (%):	3.21	***Temperature(K):***	295	***Density(g/cm^3):***	1.711

Parameters

Fragment 1

ANG1 (A) 46.709

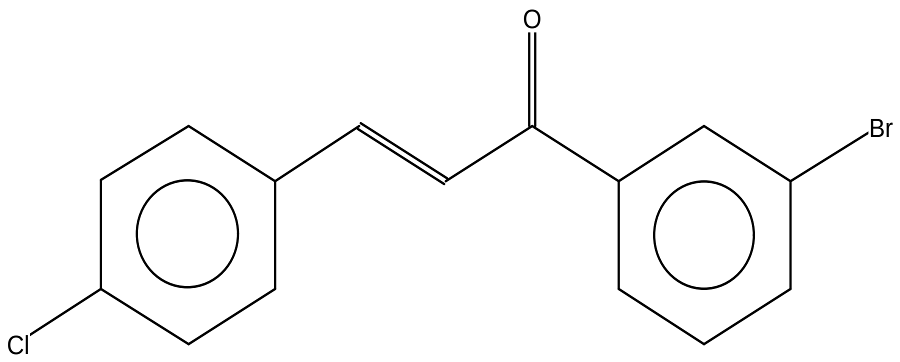

Search: search2 (Tue Jan 22 16:03:13 2019): Hit 10

VIDJIC

Reference: P.S.Patil, S.Chantrapromma, H.-K.Fun, S.M.Dharmaprakash (2007) *Acta Crystallogr.,Sect.E:Struct.Rep.Online* ,**63,**o1738

Formula: $C_{17} H_{16} Br_1 N_1 O_1$

Compound Name: 1-(3-Bromophenyl)-3-(4-dimethylaminophenyl)prop-2-en-1-one

Space Group:	Pbca	***Cell:***	***a***	13.959(0)	***b***	6.070(0)	***c***	33.350(1)
Space Group No.:	61	***(Å,°)***	α	90.00	β	90.00	γ	90.00

R-Factor (%): 4.56 ***Temperature(K):*** 295 ***Density(g/cm³):*** 1.552

Parameters

Fragment 1

ANG1 (A) 49.170

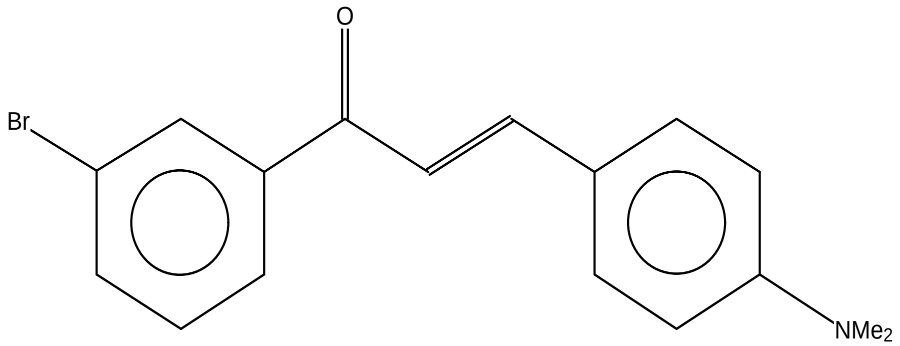

Search: search2 (Tue Jan 22 16:03:13 2019): Hit 11

XALQAF

Reference:	K.S.Harini, C.K.Quah, C.S.Chidan Kumar, S.Chandraju, N.K.Lokanath, S.Naveen, I.Warad (2017) *IUCrData* ,**2,**x170287
Formula:	C_{15} H_{10} Br_1 N_1 O_3
Compound Name:	(E)-1-(3-Bromophenyl)-3-(4-nitrophenyl)prop-2-en-1-one

Space Group:	P21	***Cell:***	***a*** 6.051(0)	***b*** 5.054(0)	***c*** 21.841(3)		
Space Group No.:	4	***(Å,°)***	α 90.00	β 95.78(0)	γ 90.00		
R-Factor (%):	3.32	***Temperature(K):***	294	***Density(g/cm³):***	1.660		

Parameters

Fragment 1
ANG1 (A) 3.318

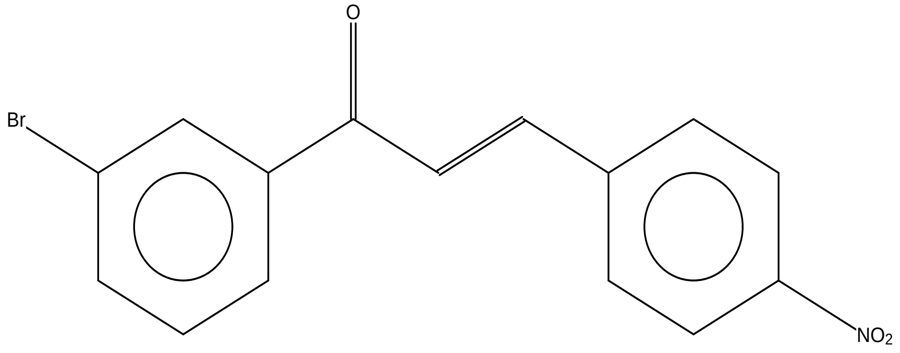

Search: search2 (Tue Jan 22 16:03:13 2019): Hit 12

ZAQCUR

Reference:	Guang-Bing Li, Lu Li, Guo-Xi Wang (2012)<br>*Acta Crystallogr.,Sect.E:Struct.Rep.Online* ,**68,**o1446
Formula:	C_{17} H_{16} Br_1 N_1 O_2
Compound Name:	(E)-1-(5-Bromo-2-hydroxyphenyl)-3-[4-(dimethylamino)phenyl]prop-2-en-1-one

Space Group:	P21/c	***Cell:***	***a*** 15.191(0)	***b***	5.450(0)	***c***	19.757(0)
Space Group No.:	14	***(Å,°)***	α 90.00	β	106.00(2)	γ	90.00
R-Factor (%):	8.89	***Temperature(K):***	123	***Density(g/cm^3):***	1.463		

Parameters

Fragment 1

ANG1 (A) 7.416

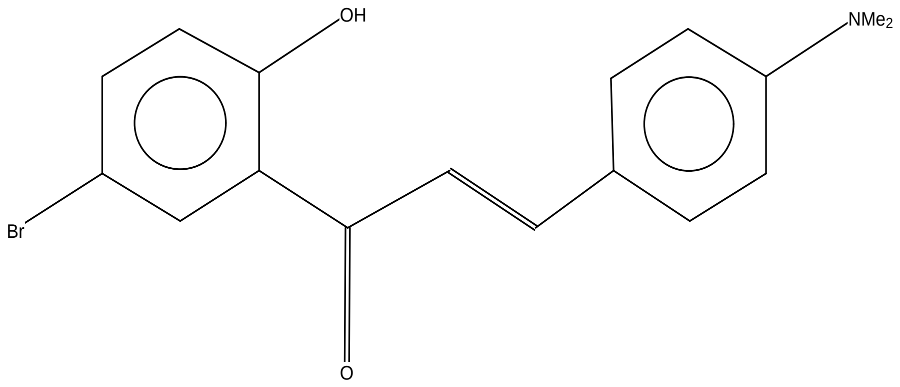

Supplement: Supplementary file 4 [file e-75-00264-sup4.pdf]
